# Room-temperature macromolecular crystallography using a micro-patterned silicon chip with minimal background scattering

**DOI:** 10.1107/S1600576716006348

**Published:** 2016-05-23

**Authors:** Philip Roedig, Ramona Duman, Juan Sanchez-Weatherby, Ismo Vartiainen, Anja Burkhardt, Martin Warmer, Christian David, Armin Wagner, Alke Meents

**Affiliations:** a Deutsches Elektronen-Synchrotron DESY, Photon Science, Notkestrasse 85, Hamburg 22607, Germany; b Diamond Light Source Ltd, Diamond House, Harwell Science and Innovation Campus, Didcot, Oxfordshire OX11 0DE, UK; c Paul Scherrer Institut, Villigen PSI, 5232, Switzerland

**Keywords:** crystallography on a chip, synchrotron serial crystallography, room-temperature crystallography, X-ray radiation damage

## Abstract

Recent success at X-ray free-electron lasers has led to serial crystallography experiments staging a comeback at synchrotron sources as well. With crystal lifetimes typically in the millisecond range and the latest-generation detector technologies with high framing rates up to 1 kHz, fast sample exchange has become the bottleneck for such experiments. A micro-patterned chip has been developed from single-crystalline silicon, which acts as a sample holder for up to several thousand microcrystals at a very low background level. The crystals can be easily loaded onto the chip and excess mother liquor can be efficiently removed. Dehydration of the crystals is prevented by keeping them in a stream of humidified air during data collection. Further sealing of the sample holder, for example with Kapton, is not required. Room-temperature data collection from insulin crystals loaded onto the chip proves the applicability of the chip for macromolecular crystallography. Subsequent structure refinements reveal no radiation-damage-induced structural changes for insulin crystals up to a dose of 565.6 kGy, even though the total diffraction power of the crystals has on average decreased to 19.1% of its initial value for the same dose. A decay of the diffracting power by half is observed for a dose of *D*_1/2_ = 147.5 ± 19.1 kGy, which is about 1/300 of the dose before crystals show a similar decay at cryogenic temperatures.

## Abstract 2

A micro-patterned sample holder of single-crystalline silicon, loaded with multiple protein crystals which are exposed to a humidified gas stream, allows high-quality room-temperature data collection.

## 1. Introduction   

Proteins are a fundamental building block of biological cells and perform a vast array of functions within living organisms. In the course of conducting their biological function, proteins in many cases undergo reversible structural changes. In order to understand these dynamics on the molecular level and their relation to molecular recognition, catalytic function and allostery, detailed descriptions of atomic coordinates and motions are desirable (Fraser *et al.*, 2011 ▸; Fenwick *et al.*, 2014 ▸). In structural biology, techniques such as X-ray crystallography, NMR spectroscopy and dual polarization interferometry are employed to investigate the atomic and molecular arrangement of proteins.

In X-ray crystallography, radiation damage limits the amount of diffraction data which can be obtained from one crystal. Radiation damage can generally be classified as either specific or global. Specific radiation damage arises from direct inelastic interaction of the X-ray photons with the sample *via* photoelectric absorption or from Compton scattering, often denoted primary damage. The subsequent radiolytic reactions are caused by the generated free electrons with energies between a few and several tens of electron volts, also referred to as secondary damage (Mozumder & Magee, 1966 ▸; Teng & Moffat, 2000 ▸; Ziaja *et al.*, 2005 ▸). Possible repercussions of specific damage are redox processes, generation of free radicals and breakage of chemical bonds, which become visible in irreversible changes of the electron-density map (Helliwell, 1988 ▸; Weik *et al.*, 2000 ▸; Meents *et al.*, 2009 ▸, 2010 ▸). Global damage, also called tertiary damage, is not specific to particular atoms and becomes apparent because of the destabilization of the crystal lattice (Henderson, 1990 ▸). Possible indications of global damage have been reported as an overall decrease in the integrated Bragg reflection intensities (especially pronounced for the high-resolution reflections), an increase in the relative *B* factors and an expansion of the unit cell (Burmeister, 2000 ▸; Ravelli & McSweeney, 2000 ▸; Kmetko *et al.*, 2006 ▸; Meents *et al.*, 2009 ▸).

At the latest low-emittance synchrotron beamlines, which can provide a photon flux of up to 10^13^ photons s^−1^ on a small spot of a few square micrometres, the crystal lifetime in the beam at room temperature is typically limited to just a few milliseconds. In comparison, for data collection at cryogenic temperatures the crystal lifetime can be extended by about two orders of magnitude to about one second (Southworth-Davies *et al.*, 2007 ▸; Burkhardt *et al.*, 2013 ▸; Garman & Owen, 2006 ▸). For this reason X-ray crystallography is typically performed at cryogenic temperatures (Haas & Rossmann, 1970 ▸; Hope, 1988 ▸; Southworth-Davies *et al.*, 2007 ▸).

However, the above-mentioned motion and dynamics in proteins can be quenched at cryogenic temperatures, meaning that the structural information obtained may not fully represent the structure of the protein in its natural environment (Rasmussen *et al.*, 1992 ▸; Tilton *et al.*, 1992 ▸). As stated by Halle (2004 ▸) in a theoretical analysis, the cryocooling process is typically too slow to fully trap the equilibrium distribution of the protein and the solvent configuration which exists at room temperature. In conformity with this perspective, Fraser *et al.* (2009 ▸) showed that catalytically essential conformations of the human proline isomerase, cyclophilin A, are populated at room temperature but not at cryogenic temperatures. Similar findings for different conformational distributions as a function of temperature were obtained for crystals of myoglobin (Frauenfelder *et al.*, 1979 ▸, 1987 ▸) and RNase (Rasmussen *et al.*, 1992 ▸; Tilton *et al.*, 1992 ▸).

Besides these structural considerations, X-ray diffraction measurements at cryogenic temperatures are also in many cases associated with practical problems concerning crystal handling and cryo-protection. Cryo-protectants such as ethylene glycol and glycerol are normally added to protect the sample crystals from freezing damage, *e.g.* due to the formation of hexagonal ice. Upon cryo-protection, the native environment of the crystals is changed. In particular, membrane proteins, viruses and other large-unit-cell systems tend to yield small and fragile crystals which are very sensitive to temperature variations. Here, cryo-protection procedures can be tedious and often have a detrimental effect on crystal quality and their diffracting properties (Axford *et al.*, 2012 ▸). One method which allows sample vitrification without cryo-protectants is high-pressure freezing, where samples are cryo-cooled while being subjected to high pressures of several hundreds of megapascals (Thomanek *et al.*, 1973 ▸; Kim *et al.*, 2005 ▸; Burkhardt *et al.*, 2012 ▸, 2013 ▸).

With the development of X-ray free-electron lasers (XFELs) in recent years, crystallography experiments at room temperature are staging a comeback. At XFELs, a single diffraction pattern of a crystal is recorded before the crystal is vaporized by the intense X-ray pulse, an approach often denoted ‘diffraction before destruction’ (Chapman *et al.*, 2011 ▸). With the availability of new high-frame-rate integrating detectors (Philipp *et al.*, 2011 ▸; Herrmann *et al.*, 2013 ▸; Jungmann-Smith *et al.*, 2014 ▸), this has led to the emerging field of ‘serial crystallography’, where diffraction patterns of hundreds to hundreds of thousands of crystals are measured in a reasonable time and the few recorded reflections from each individual crystal are subsequently merged to give a complete data set (Boutet *et al.*, 2012 ▸; Neutze & Moffat, 2012 ▸; Redecke *et al.*, 2013 ▸; Hunter *et al.*, 2014 ▸). Performing these experiments at room temperature allows, in addition to static structure determination, the investigation of protein kinetics, *e.g.* enzyme reactions (Kupitz *et al.*, 2014 ▸; Tenboer *et al.*, 2014 ▸).

Originally devised for crystallography experiments at XFELs, this serial approach has recently also been successfully employed at synchrotron sources. In particular, the development of high-frame-rate single-photon counting detectors has allowed the measurement of tens of thousands of diffraction patterns in a reasonable time at synchrotrons (Broennimann *et al.*, 2006 ▸; Pennicard *et al.*, 2012 ▸).

Different sample-delivery techniques such as liquid jets (Stellato *et al.*, 2014 ▸; Botha *et al.*, 2015 ▸), fixed targets (Zarrine-Afsar *et al.*, 2012 ▸; Gati *et al.*, 2014 ▸; Mueller *et al.*, 2015 ▸; Murray *et al.*, 2015 ▸; Roedig *et al.*, 2015 ▸; Baxter *et al.*, 2016 ▸), and array-like plates for *in situ* crystal growth and diffraction (McPherson, 2000 ▸; Kisselman *et al.*, 2011 ▸; Axford *et al.*, 2012 ▸; Pinker *et al.*, 2013 ▸; Heymann *et al.*, 2014 ▸; Huang *et al.*, 2015 ▸) have been successfully used at synchrotrons. The challenges of the currently applied sample-delivery techniques are the high crystal consumption and the high background scattering levels caused by the surrounding mother liquor in the case of liquid jets. The use of ‘toothpaste’-like lipidic cubic phase jets can significantly reduce the crystal consumption (Weierstall *et al.*, 2014 ▸) but is accompanied by a high background scattering level caused by the lipidic crystal matrix of the jet. Particularly in the case of smaller crystals with sizes of only a few micrometres, the weak diffraction signals are often buried in the background.

A promising approach is the use of fixed substrates which allow for efficient removal of the surrounding mother liquor and therefore generate almost no background (Roedig *et al.*, 2015 ▸). The chips developed by our group are made of single-crystalline silicon so that, in a diffraction experiment, the substrate itself only contributes with distinct Bragg reflections from the silicon, which are typically at higher resolution shells than the protein diffraction and can be avoided by exact knowledge of the orientation of the substrate.

A significant challenge when performing macromolecular crystallography experiments with fixed substrates at room temperature is to prevent dehydration of the crystals after removal of the mother liquor. In order to ensure a constantly humid environment during data collection, a possible solution is to place the chip in a sealed container, using for example Mylar foil (Mueller *et al.*, 2015 ▸; Sherrell *et al.*, 2015 ▸). However, these approaches may be unfavourable with respect to the scattering background contribution of the Mylar foil and the additional tedious preparation step which becomes necessary.

In the following, we present a novel approach for room-temperature data collection from multiple macromolecular crystals mounted on a chip bathed in an open stream of humidified air. The humidified air stream, with adjustable relative humidity, is provided by a humidity control device (Sanchez-Weatherby *et al.*, 2009 ▸). By this means the crystals can be kept stable for several hours without losing their diffracting properties owing to dehydration. Additionally, this technique enables experiments including controlled dehydration of protein crystals, which has been shown to improve the diffraction qualities of the crystals in many cases (Kiefersauer *et al.*, 2000 ▸; Umena *et al.*, 2011 ▸). Furthermore, reactions inside the protein crystals can be initiated by changing the chemical composition of the vapour stream (Sjögren *et al.*, 2002 ▸).

In order to prove this concept and the applicability of our chip to room-temperature serial crystallography, we have performed room-temperature data collection from multiple cubic insulin crystals loaded onto our chip. It was a further goal of the present work to investigate specific and global radiation damage effects at room temperature as a function of dose and to draw conclusions from this for future room-temperature serial crystallography experiments at synchrotron sources.

## 2. Materials and methods   

### 2.1. Sample preparation and chip handling   

Porcine insulin was purchased from Sigma–Aldrich (catalogue No. I-5523). Cubic Zn-free crystals of porcine insulin were grown from alkaline sodium phosphate buffer by hanging-drop vapour diffusion (Jackson, 1974 ▸). A solution of 15 or 30 mg ml^−1^ insulin in Na_2_HPO_4_ buffer (*c*_Phos_ = 0.05 *M*, 0.01 *M* EDTA, pH 9.8–10.4) was mixed with Na_2_HPO_4_ buffer (*c*_Phos_ = 0.4–0.6 *M*, 0.01 *M* EDTA, pH 9.8–10.4) at different ratios (1:3, 1:1, 2:1, 3:1). The overall drop volume was adjusted to 6 µl. The mixture was equilibrated against 750 µl of Na_2_HPO_4_ buffer (*c*_Phos_ = 0.4–0.6 *M*, 0.01 *M* EDTA, pH 9.8–10.4) at 298 K. Insulin microcrystals between 20 and 50 µm in size were obtained after 3–5 h.

**Figure 1:**
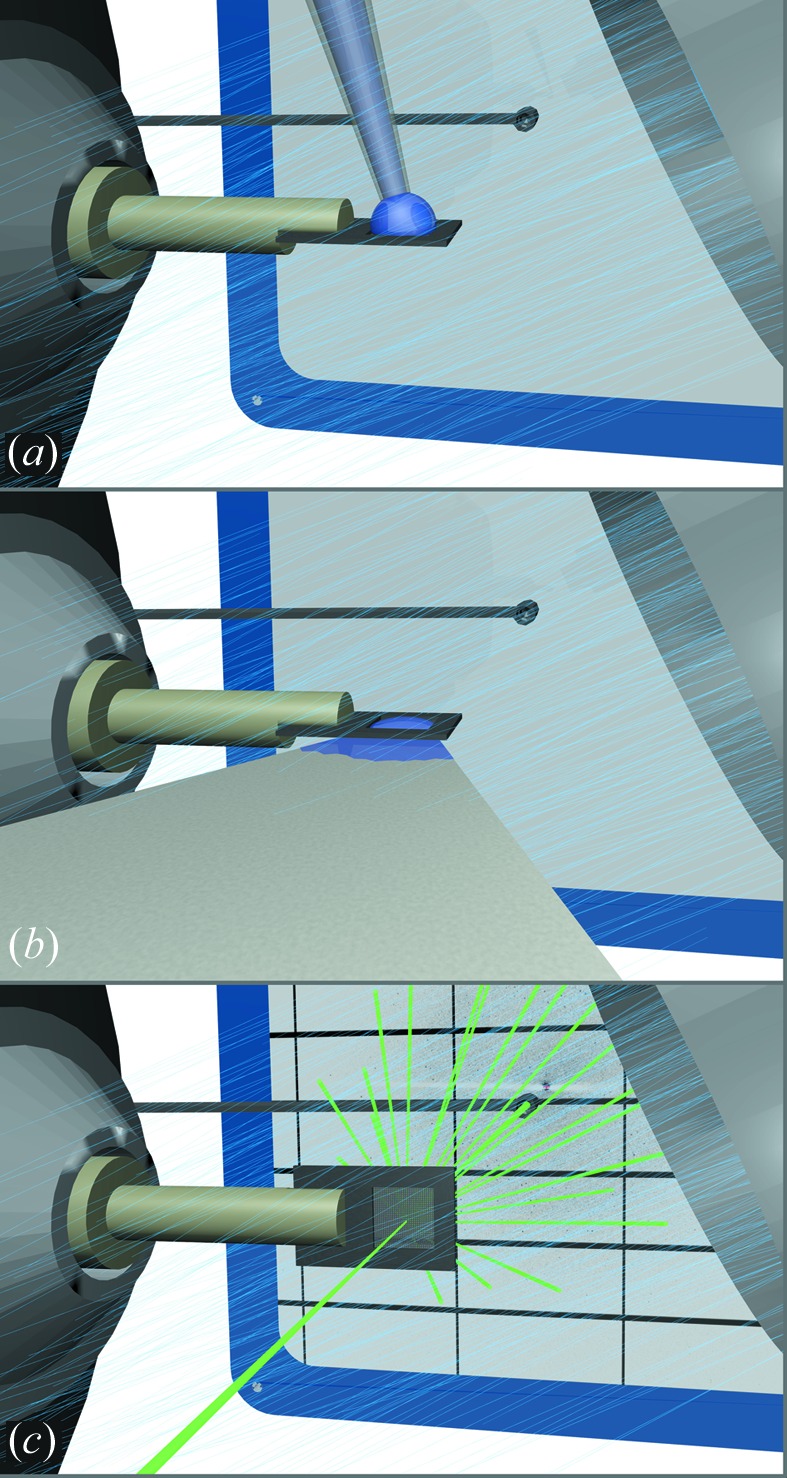
Schematic illustration of the experimental handling. The silicon chip is mounted directly on the beamline goniometer and positioned within a stream of humidified air. (*a*) A drop of 2–3 µl of crystal suspension is pipetted onto the upper side of the chip. (*b*) By attaching a wedge of filter paper to the bottom side of the chip, the mother liquor is then soaked through the pores while the crystals are retained by the chip. (*c*) Finally, the microcrystals are scanned through the primary beam. The diffraction pattern is recorded with a flat Pilatus 6M detector. Dehydration of the crystals is prevented by the humidified air.

**Figure 2:**
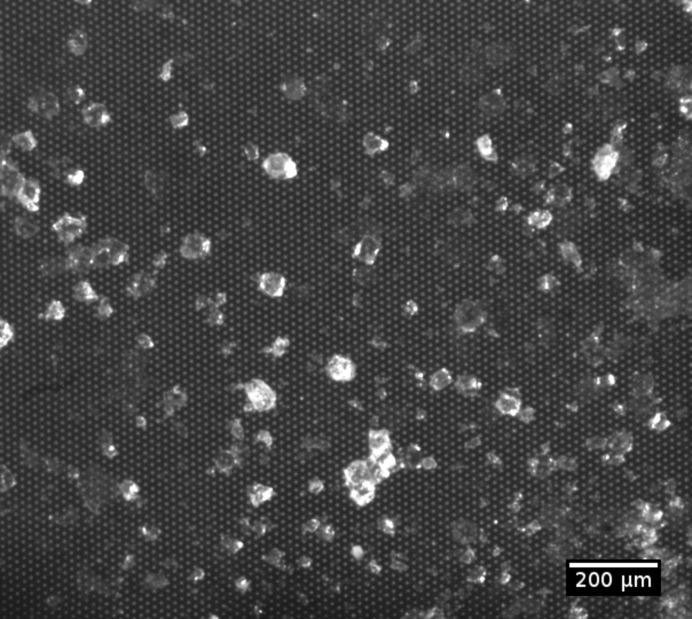
Microscope image of the insulin crystals distributed over the chip membrane. The micropores are 8 µm in diameter and are arranged in a triangular grid.

A micro-patterned silicon chip was used as a sample holder, similar to the device recently described by our group for data collection at cryogenic temperatures (Roedig *et al.*, 2015 ▸). The chip itself was attached to a standard magnetic base, which was directly mounted on the beamline goniometer as shown in Fig. 1 ▸. To prevent dehydration of the crystals during the experiment, the chip was continuously exposed to a stream of humidified air. For this, a humidity control device, HC1 (Arinax, France), was installed on the beamline goniometer, and it provided a continuous flow of 4 l min^−1^ humidified air with adjustable relative humidity (Sanchez-Weatherby *et al.*, 2009 ▸). During data collection, the relative humidity was set to the highest possible value (∼100%) and was probably between 98 and 99% at the sample position. For crystal loading, a drop of 2–3 µl of the crystal suspension was pipetted onto the chip (Fig. 1 ▸ *a*). Subsequently, the mother liquor was blotted off the back of the chip through the micropores of the silicon membrane using a wedge of blotting paper (Fig. 1 ▸ *b*). The diameter of the pores was chosen to be ∼10 µm so that insulin crystals larger than the pore diameter were retained, while the surrounding mother liquor and other solid material smaller than the pore diameter were efficiently removed. A microscope image of insulin crystals loaded onto the chip is shown in Fig. 2 ▸. It can be clearly seen that the crystals are surrounded by only very small amounts of mother liquor.

### 2.2. Data collection   

X-ray data collection was performed on the macromolecular beamline I03 at Diamond Light Source. An X-ray beam with a size of 50 × 50 µm (FWHM) and a photon flux of 5.7 × 10^10^ photons s^−1^ at an X-ray energy of 12.36 keV was used for the experiment. Individual crystals were selected and centred in the X-ray beam using the on-axis microscope installed on the beamline. From every crystal, 700 diffraction images, with a rotation increment of 0.1° and an exposure time of 0.1 s per image, were collected using a Pilatus 6M detector, resulting in a total rotation range of 70° and a total exposure time of 70 s per data set. Owing to the small thickness of the silicon membrane, typically less than 10 µm, the X-ray absorption by the chip at the given energy is less than 4% for normal incidence. With crystal dimensions of about 30 × 30 × 30 µm and a solvent content of 60%, the average absorbed X-ray dose for each recorded diffraction image was calculated as 0.808 kGy, using *RADDOSE-3D* (Zeldin *et al.*, 2013 ▸).

### 2.3. Data processing and phasing   

The recorded data sets were indexed and integrated using *XDS* (Kabsch, 2010*b* ▸). In order to study radiation damage effects, each data set from a crystal was divided into 14 subsets of 50 frames for data quality analysis and into seven subsets of 100 frames for structure refinement. Partial data sets from different crystals were merged using *XSCALE* (Kabsch, 2010*b* ▸) to obtain a complete data set for each subset. Subsets from five different insulin crystals with 100 frames each were sufficient for a complete data set, owing to the high symmetry of cubic insulin (space group *I*2_1_3). Since this space group is one of the 27 space groups for protein crystals in which the symmetry of the Bravais lattice is higher than the symmetry of the space group itself, an indexing ambiguity with two possible indexing modes exists (Brehm & Diederichs, 2014 ▸). As a consequence, data sets from about half of the crystals had to be reindexed before merging. By maximizing the Pearson correlation coefficient
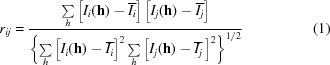
between the individual partial data sets *i* and *j* with intensities *I_i_* and *I_j_* of common reflections **h** = (*h*,*k*,*l*), complete and consistent data sets with correlation coefficients *r_ij_* ≃ 1 were obtained.

Structure solution was performed by molecular replacement with *Phaser* (McCoy *et al.*, 2007 ▸), using PDB model 9ins as a template (Gursky *et al.*, 1992 ▸), and the structure was refined isotropically with *phenix.refine* (Adams *et al.*, 2010 ▸). This procedure was performed for each subset. Refinement statistics for the first subset (absorbed dose 80.8 kGy) and the last subset (absorbed dose 565.6 kGy) are summarized in Table 1 ▸. The refined structure for the first subset (absorbed dose 80.8 kGy) is available under PDB code 5fb6. In addition, raw images and processing results for all subsets can be downloaded from http://zenodo.org using the doi 10.1107/S1600576716006348.

## 3. Results and discussion   

### 3.1. Integrated data statistics   

**Figure 3:**
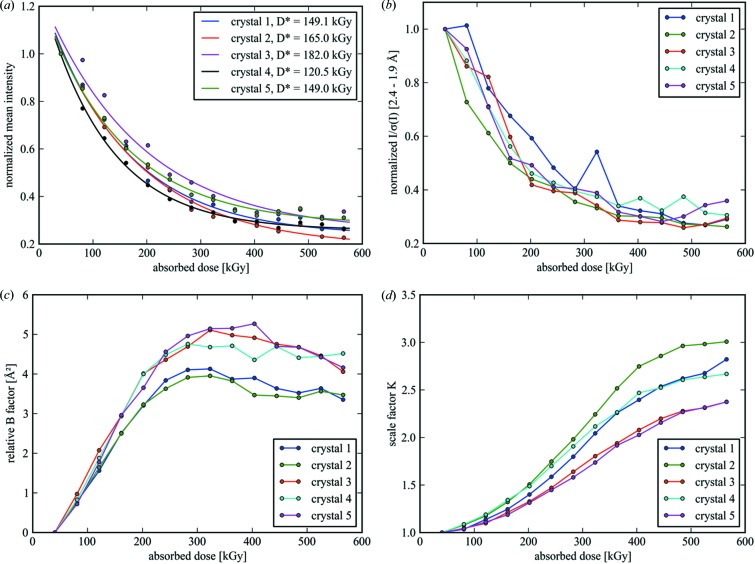
Diffraction data-quality parameters as a function of dose for five insulin crystals measured at room temperature, showing clear manifestations of radiation damage. (*a*) The decay of the mean intensities of the whole data sets, (*b*) the *I*/σ(*I*) ratio for reflections in the high-resolution shell 2.4–1.9 Å (normalized to the value of the first subset), and the increase in (*c*) the relative *B* factors and (*d*) the scaling factors, as defined by equation (3), are all plotted as a function of absorbed dose. The solid lines in part (*a*) correspond to an exponential fit as given by equation (2), while the lines in parts (*b*)–(*d*) are meant as a guide to the eye.

The decay of different diffraction data quality indicators as a function of dose is shown in Fig. 3 ▸. The mean intensity over the whole resolution range (Fig. 3 ▸ *a*) and the *I*/σ(*I*) ratio of the high-resolution reflections (Fig. 3 ▸ *b*) show a distinct loss of signal as determined by *XDS* processing. While for data collection at cryo-temperatures a linear correlation model between damage rate and absorbed dose has been found to be appropriate in many cases (Teng & Moffat, 2000 ▸; Diederichs *et al.*, 2003 ▸), both quadratic and exponential approaches have also been proposed (Burmeister, 2000 ▸; Diederichs, 2006 ▸). As shown in Fig. 3 ▸(*a*), the mean intensity for each crystal is well described by an exponential decay in the form
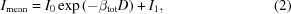
where *D* is the absorbed dose, β_tot_ the damage rate constant, *I*_0_ the fraction of the overall intensity which is directly affected by radiation damage effects and *I*_1_ a constant term which accounts for an undamaged fraction in the crystal. For ease of comparability, the values of *I*_mean_ were normalized to the values of the first subset, corresponding to an absorbed dose of *D* = 40.4 kGy. We define the specific dose as D* = 1/β_tot_, which is a measure of the amount of dose a crystal can absorb until the first term in equation (2) is reduced to 1/*e* times its initial value *I*_0_. Fitted parameters for five chosen insulin crystals, obtained using the nonlinear least-squares method, are given in Table 2 ▸. By this means an average specific dose of D* = 153.1 ± 22.8 kGy is obtained for the insulin crystals in our experiment. A similar decay is observed for the intensities *I*/σ(*I*) of the high-resolution reflections [Fig. 3 ▸(*b*), also normalized to the value of the first subset]. The values for the mean intensity do not vanish completely, but converge to a lower boundary represented by the fitting parameter *I*_1_ in the dose range under consideration. The values of this constant term *I*_1_ are on average 19.1% of the theoretical initial mean intensity *I*_0_ + *I*_1_ for the undamaged crystal, which corresponds to *D* = 0 in equation (2). This behaviour is seen for all considered crystals. Since the beam was typically larger than the crystals and the crystals were therefore completely immersed in the X-ray beam, this observation cannot be attributed to new unexposed crystalline material which could have been rotated into the beam during oscillation. As suggested by Blake & Phillips, the observations indicate a fraction of crystalline structure which seemed to be rather unaffected by radiation damage processes, *i.e* with a damage rate constant much larger than the values obtained for β_tot_ (Blake & Phillips, 1962 ▸; Hendrickson, 1976 ▸).

Besides the above-mentioned parameters, an interesting quantity is the dose required to reduce the mean intensity from its initial value *I*_0_ + *I*_1_ by half to (*I*_0_ + *I*_1_)/2, denoted *D*_1/2_. Its relation to the above-mentioned fit parameters is given by *D*_1/2_ = D*ln[2*I*_0_/(*I*_0_ − *I*_1_)]. As shown in Table 2 ▸ for the insulin crystals under study, this value amounts to *D*_1/2_ = 147.5 ± 19.1 kGy on average. This can be compared with the value of *D*_1/2_ = 43 MGy for cryogenic temperatures, which was observed by Owen *et al.* (2006 ▸) from diffraction experiments with holoferritin and apoferritin crystals. From this, we conclude that the crystals, when measured at room temperature, can tolerate only 1/292 of the applied dose before showing a similar decay in their diffracting power in comparison to cryogenic temperatures.

It is noteworthy that the onset of the decay in the diffracting power occurs right from the beginning as the crystal is exposed to the applied X-ray radiation, which can be attributed to the relatively low dose rate of 16.2 kGy s^−1^. Our observation is consistent with the results shown by Owen *et al.* (2014 ▸), who described the loss in diffracting power as a function of dose with an initial slow decay, a so-called lag phase, followed by a faster exponential decay. However, those authors showed that the lag phase, lasting up to 500 kGy, is only observed for very high dose rates (>1 MGy s^−1^) and becomes absent for smaller dose rates. It turns out that, at room temperature and higher dose rates, dynamic effects become apparent which are governed by the diffusion of free radicals through the crystal lattice, particularly hydroxyl (OH^−^), the quenching of radicals by solvent molecules and free-radical recombination.

The dose dependence of the relative isotropic *B* factor can be taken as another indicator of global damage (Kmetko *et al.*, 2006 ▸; Meents *et al.*, 2009 ▸). Relative *B* factors as given by *XSCALE* [see Kabsch (2010*a* ▸) for details] are plotted in Fig. 3 ▸(*c*) for each crystal. The values were obtained by scaling all subsets from an individual crystal to the first subset. By this means, all subsets from the same crystal are placed on the same scale and the scaled intensities *I*_scaled_(**h**) are given by
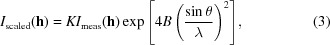
where *I*_meas_(**h**) is the measured intensity, *B* the relative *B* factor and *K* an additional absolute scale factor. Since all subsets were scaled to the corresponding first subset of each crystal, the values are *K* = 1 and *B* = 0 for the first subset. As shown in Fig. 3 ▸(*c*), a linear increase in the relative *B* factor is observed in the dose range 40–200 kGy. At higher doses, the relative *B* factor reaches a saturation level before it decreases back to smaller values. As shown in Fig. 3 ▸(*d*), for the scale factor *K* a monotonic increase is observed which is consistent with the overall decrease in the diffraction intensity.

### 3.2. Structure refinement statistics   

**Figure 4:**
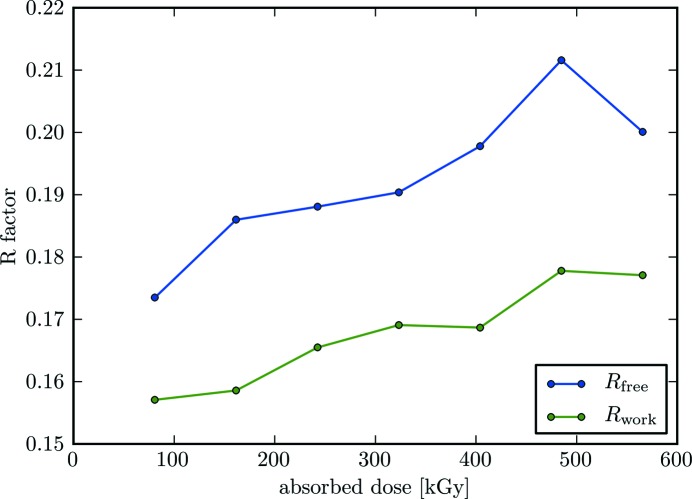
The *R* values *R*_work_ and *R*_free_ from structure refinement, plotted as a function of dose. For each subset, corresponding to a certain absorbed dose, partial data sets from crystals 1–5 were merged to give a complete data set and then refined. Merged data sets were refined in the overall resolution range 30–1.9 Å.

The refinement *R* values of the merged data sets are shown in Fig. 4 ▸. In general, an increase in the refinement *R* values is observed with respect to the X-ray dose absorbed by the crystal. For the first subset, corresponding to an absorbed dose of 80.8 kGy, high-quality electron-density maps with model *R* values of *R*_work_/*R*_free_ = 15.71/17.35 could be obtained from the merged data sets. With increasing dose these values increase to *R*_work_/*R*_free_ = 17.71/20.01, which were obtained from refinement of the last subset. Complete refinement statistics of the merged data from the first and last subsets are provided in Table 1 ▸.

### 3.3. Site-specific damage   

**Figure 5:**
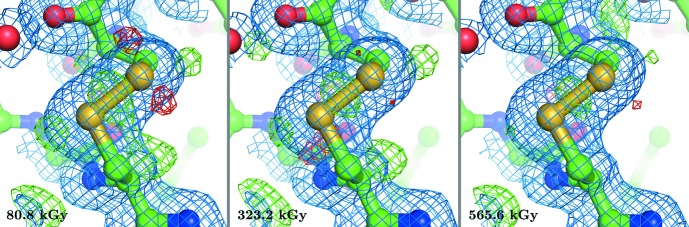
Electron-density maps of the disulfide bond between residues CysA7 and CysB7 for various absorbed doses. Electron-density maps 2*mF*_o_ − *DF*_c_ (blue) are shown at the 1σ sigma level and electron-density difference maps *mF*_o_ − *DF*_c_ (green/red) at 3σ. No indications of specific radiation damage are visible.

The disulfide bridges in insulin crystals are known to be sensitive to radiation damage. In particular, the di­sulfide bond between residues CysA7 and CysB7 has been reported to be highly affected by specific radiation damage in the range 30–60 MGy at cryogenic temperatures between 5 and 100 K (Meents *et al.*, 2010 ▸). However, such changes in the electron-density map could not be observed in the present experiments at room temperature. As shown in Fig. 5 ▸, no indications of specific radiation damage for this di­sulfide bond could be observed up to an absorbed dose of 565.6 kGy. Careful inspection of the electron-density maps and atomic *B* factors did not reveal any other sites of specific radiation damage for the overall dose applied.

## 4. Conclusions   

Our chip is very well suited to room-temperature data collection from multiple crystals from a few micrometres up to several tens of micrometres in size. Loading of the crystals onto the chip is straightforward and takes less than a minute. The crystal suspension is pipetted onto the chip and the mother liquor is blotted from the reverse through the pores using a wedge of filter paper. Owing to the sieve-like structure of the chip, all crystals larger than the pore size are retained after blotting and can be utilized for data collection. Since single-crystalline silicon is used as the chip material, the background contribution of the chip itself is negligible. Dehydration of the crystals is prevented by exposing them to a stream of humidified air during sample loading and data collection. This approach requires no further sealing with additional material such as Kapton or Mylar foil which would lead to an increased background level. Using humidified helium gas instead of air would further decrease the path the scattered X-rays need to pass in air, thus further reducing the background level.

High-quality X-ray diffraction data sets merged from the measurement of several crystals could be obtained and were analysed with respect to potential radiation damage effects. The diffracting power of the insulin crystals decreases exponentially as a function of the applied X-ray dose and reaches 1/*e* of its initial value already after a dose of 153.1 ± 22.8 kGy. However, in contrast with the overall reduction in diffraction power, no signs of specific radiation damage could be observed from the electron-density maps for doses up to 565.6 kGy. An explanation for this different behaviour between global and specific radiation damage could be that specific radiation damage – and here in particular cleavage of disulfide bridges – is less temperature dependent than global radiation damage and generally occurs only at higher doses. This means that disulfide bond breakage is not the preferred damage pathway at room temperature, where global radiation damage to the lattice is clearly the dominating effect.

All five insulin crystals show a similar decay of the diffraction quality parameters *versus* dose, with small variations between the different crystals. This further highlights the applicability of our chip in combination with a humidified gas stream for a room-temperature crystallography setup which provides identical measurement conditions for all crystals. Previous room-temperature crystallography experiments were mostly carried out with crystals mounted in glass capillaries. For this, all crystals had to be individually soaked into the capillaries, which subsequently had to be sealed with hot wax to prevent solvent evaporation. Using this approach, crystals often suffered from dehydration and heating effects, which led to a large spread of the diffraction quality parameters between individual crystals.

In summary, our chip has been proven very useful for room-temperature data collection from multiple crystals. Sample preparation is straightforward, since the loading of thousands of crystals onto the chip takes less than a minute. With respect to macromolecular X-ray crystallography experiments at cryogenic temperatures, we observed that crystals can tolerate 1/292 of the dose before showing a similar decay in the diffracting power. This means that, at room temperature, about 300 times the number of crystals have to be measured in order to obtain a diffraction data set of similar quality to that at cryogenic temperature.

In the present experiment the crystals were relatively large, which allowed us to collect data in large rotation increments from every crystal. In the case of smaller crystals and aiming for the same resolution, because of radiation damage effects only data from smaller rotation increments can be collected. Hence, for structure determinations, data collection from a larger number of crystals will be required. For very small crystals in the range of a few micrometres, fast raster-scanning of the chip without any rotation might become the method of choice here.

We expect that such room-temperature measurements with microcrystals loaded onto our chip will also be well suited for measurements at XFELs and for future kinetic investigations of irreversible enzyme reactions.

## Supplementary Material

PDB reference: porcine insulin, 5fb6

Raw data link, http://zenodo.org URL: http://zenodo.org

## Figures and Tables

**Table 1:** Diffraction data and refinement statistics for the first and last merged subsets, corresponding to absorbed doses of 80.8 and 565.6 kGy, respectively Values in parentheses are for the highest-resolution shell.

	80.8 kGy	565.6 kGy
X-ray wavelength (Å)	1.003	1.003
Resolution range (Å)	30–1.9 (2.0–1.9)	30–1.9 (2.0–1.9)
Unit-cell lattice constant	78.8 Å (cubic)	78.8 Å (cubic)
Space group	*I*2_1_3	*I*2_1_3
Total reflections	33764 (3372)	34346 (3349)
Unique reflections	6542 (652)	6480 (640)
Multiplicity	5.2 (5.2)	5.3 (5.2)
Completeness (%)	99.21 (99.69)	98.89 (98.77)
Mean *I*/σ(*I*)	18.04 (5.16)	10.34 (1.25)
CC_1/2_	0.999 (0.918)	0.996 (0.375)
CC*	1.0 (0.978)	0.999 (0.738)
Wilson *B* factor (Å^2^)	24.58	24.58
*R*_merge_	0.05801 (0.372)	0.1474 (1.327)
*R*_meas_	0.06457	0.1632
*R*_work_/*R*_free_	15.71/17.35	17.71/20.01
No. of non-hydrogen atoms	474	479
Macromolecules	451	451
Water molecules	23	28
Protein residues	51	51
Average *B* factor (Å^2^)	29.30	28.90
Protein	28.70	28.20
Solvent	41.80	40.30
R.m.s. deviation, bonds (Å)	0.009	0.009
R.m.s. deviation, angles (°)	0.93	0.94
Ramachandran plot (%)		
Favoured	98	98
Allowed	2	2
Outliers	0	0
Clashscore	4.51	6.76

**Table 2:** Fit parameters corresponding to equation (2) for the five insulin crystals shown in Fig. 3 ▸

Crystal	*I*_0_	*I*_1_	β_tot_ (kGy^−1^)	D* = 1/β_tot_ (kGy)	*I*_1_/(*I*_0_ + *I*_1_)	*D*_1/2_ = D*ln[2*I*_0_/(*I*_0_ − *I*_1_)]
Crystal 1	1.04 ± 0.04	0.236 ± 0.019	0.0067 ± 0.0006	149.1 ± 12.7	0.185 ± 0.013	141.7 ± 12.7
Crystal 2	1.06 ± 0.03	0.188 ± 0.018	0.0061 ± 0.0004	165.0 ± 11.4	0.150 ± 0.012	146.5 ± 10.7
Crystal 3	1.02 ± 0.05	0.249 ± 0.042	0.0055 ± 0.0008	182.0 ± 28.0	0.196 ± 0.028	177.0 ± 29.2
Crystal 4	1.03 ± 0.02	0.257 ± 0.006	0.0083 ± 0.0003	120.5 ± 4.2	0.199 ± 0.005	118.0 ± 4.3
Crystal 5	0.97 ± 0.03	0.280 ± 0.013	0.0067 ± 0.0004	149.0 ± 9.3	0.225 ± 0.009	154.3 ± 10.2
